# A discussion of cryo-EM terminology as the outreach and number of PDB entries expand

**DOI:** 10.1107/S2052252526007505

**Published:** 2026-09-01

**Authors:** Bruno P. Klaholz

**Affiliations:** a Centre for Integrative Biology (CBI), Department of Integrated Structural Biology, IGBMC (Institute of Genetics and of Molecular and Cellular Biology), 1 rue Laurent Fries, Illkirch, France; b Centre National de la Recherche Scientifique (CNRS) UMR 7104 Illkirch France; c Institut National de la Santé et de la Recherche Médicale (Inserm) U964 Illkirch France; d Université de Strasbourg Strasbourg France; MRC Laboratory of Molecular Biology, United Kingdom

**Keywords:** 3D reconstruction and image processing, cryo-EM, cryo-ET, Protein Data Bank, PDB

## Abstract

Cryo electron microscopy (cryo-EM) has made great advances in the last decade, progressively increasing its impact in structural biology as a key method to address molecular structures and mechanisms of various macromolecular complexes. Single-particle cryo-EM will soon equal the number of yearly entries in the Protein Data Bank from structures determined by X-ray crystallography. This is largely thanks to improved cryo electron microscope instrumentation and advanced image-processing tools and structure-sorting methods. As the role of cryo-EM is expanding to an increasingly large community, including newcomers and scientists joining from related fields, it is timely to revisit some fundamental concepts and basics of single-particle cryo-EM and image processing as terminology has become less well defined and, in some cases, confusing. Here we summarize and define some typical terms important for understanding the underlying physical concepts. These include ‘cryo-EM’, ‘cryo-ET’, ‘3D reconstruction’, ‘coarsening’, ‘contour level’ and others. We also discuss resolution estimation and map deposition.

## Abstract 2

Single-particle cryo-EM will soon equal the number of yearly entries in the Protein Data Bank from structures determined by X-ray crystallography. Considering the growing outreach of cryo-EM, it is timely to remind users of and define typical terms important for understanding the underlying fundamental physical concepts and key basics of single-particle cryo-EM and image processing, which are particularly relevant for the expanding cryo-EM community, including for newcomers and scientists joining from related fields.

## 1. Introduction

**Figure 1:**
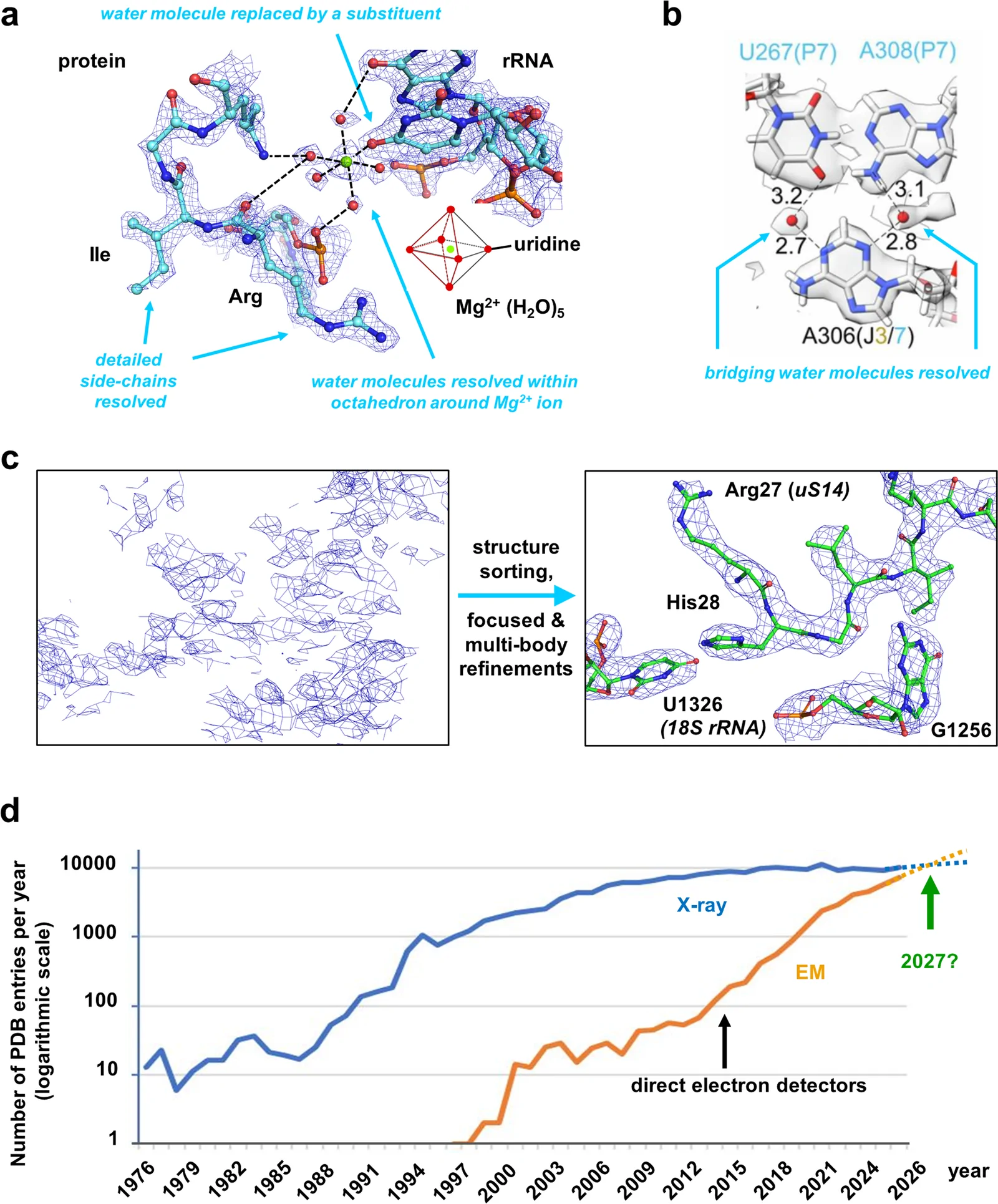
Increasing outreach of cryo-EM in terms of high-resolution structural analysis, structure sorting and data depositions. (*a*) Example of high-resolution cryo-EM enabling detailed analysis of molecular interactions of protein–RNA complexes, resolving Mg^2+^ ions and individual associated water molecules (Holvec *et al.*, 2024 ▸, https://doi.org/10.1038/s41594-024-01274-x; adapted with permission). (*b*) Example of localization of water molecules in an RNA complex, which play a bridging role (Kretsch *et al.*, 2025 ▸, https://doi.org/10.1038/s41586-025-08855-w; reproduced with permission). (*c*) Example of structure sorting thanks to advanced image processing, allowing different structural states and improved resolution to be obtained (Barchet *et al.*, 2023 ▸, https://doi.org/10.1016/j.jsb.2023.108015; reproduced with permission). (*d*) Annual PDB deposition of structures derived from X-ray crystallography and cryo-EM as of December 31st 2025 (https://www.rcsb.org/statistics/growth/experimental-method). From the current statistics, it can be expected that the annual contributions will become equivalent at the end of 2026 or during 2027 and that cryo-EM depositions will continue to increase subsequently.

Over the last decade, cryo electron microscopy (cryo-EM) of isolated complexes, together with cryo electron tomography (cryo-ET) at the cellular level, has revolutionized the way molecular mechanisms can be analysed and has become a cornerstone of structural biology even more than before. Cryo-EM has, for example, accelerated drug discovery by revealing the atomic structures of drug targets, including membrane proteins, viruses (SARS-CoV-2 and others), ribosomes and other macromolecular machines, deepening our understanding of cellular processes and disease mechanisms thanks to high-resolution structural analysis [Figs. 1 ▸(*a*) and 1 ▸(*b*)]. Technological advancements, such as improved detectors, automated data collection and advanced computational algorithms, have further enhanced the resolution and accessibility of cryo-EM. This is largely thanks to improved cryo electron microscope instrumentation (in particular, direct electron detectors) and advanced image-processing tools, including methods to analyse different structural states [Fig. 1 ▸(*c*)]. As a result, cryo-EM has become a cornerstone of modern structural biology, fostering interdisciplinary collaborations and opening new avenues for scientific discovery. Single-particle cryo-EM will soon equal the number of yearly entries in the Protein Data Bank (PDB) from structures determined by X-ray crystallography, which is anticipated to happen at the end of 2026 or in 2027 [Fig. 1 ▸(*d*)]. Yet, cryo-EM and X-ray crystallography should not be understood as competing methods but indeed are very complementary. Once diffracting crystals have been obtained, X-ray crystallography is usually easier and faster to perform for comparisons of related structural states such as screening ligand complexes, comparing different DNA sequences in a nucleoprotein complex or analysing structural variants such as point mutations. Cryo-EM does not need crystallization though and is often applied to medium and large complexes [see PDB statistics by Klaholz (2019 ▸)], but even smaller complexes are being analysed by cryo-EM. However, many concepts (Fourier transforms, importance of phases, masking/solvent flattening *etc*.) show significant similarities, and a knowledge of one of the techniques can quickly be applied to the other. Also, as this special issue highlights, there is much to improve at different levels in cryo-EM and cryo-ET, from sample preparation, instruments for data acquisition, image processing and data analysis, atomic model building – also in combination with structure prediction and prediction/refinement tools such as *AlphaFold2/3* (Abramson *et al.*, 2024 ▸), *RosettaFold* (Baek *et al.*, 2021 ▸), *Phenix* (Terwilliger *et al.*, 2023 ▸) and others (benefitting from 50 years of structure depositions since the creation of the PDB in 1976) – to structure interpretation.

In the last few years, cryo-EM has increased its outreach in the literal sense of serving communities, including those not having access to these services due to the costly high-end instrumentation required. Many infrastructures have been built such as Instruct-ERIC at the European level (https://instruct-eric.org/), eBIC in the UK or the NIH-based Cryo-EM Centres in the US (https://www.cryoemcenters.org/). They offer access to high-end instrumentation, expertise and training. While the past decade has firmly established cryo-EM as an indispensable tool and has helped to shape the future of biomedical research and therapeutic innovation, the strong growth and wide applicability has also led to a tendency to neglect the underlying concepts at instrumentation, sample preparation and image-processing levels including 3D reconstruction and structure refinement. Terminology has also sometimes become less well defined, leading to wordings being inadvertently used in a confusing manner, for example within publications, software and tools therein, during courses and lectures or in student reports and PhD theses. In the following, we refresh and define typical terms important for understanding the underlying fundamental physical concepts and key basics of single-particle cryo-EM and image processing, some of which apply to both cryo-EM and cryo-ET, which are particularly relevant for the expanding cryo-EM community, including newcomers and scientists joining from related fields.

## 2. Cryo-EM

**Figure 2:**
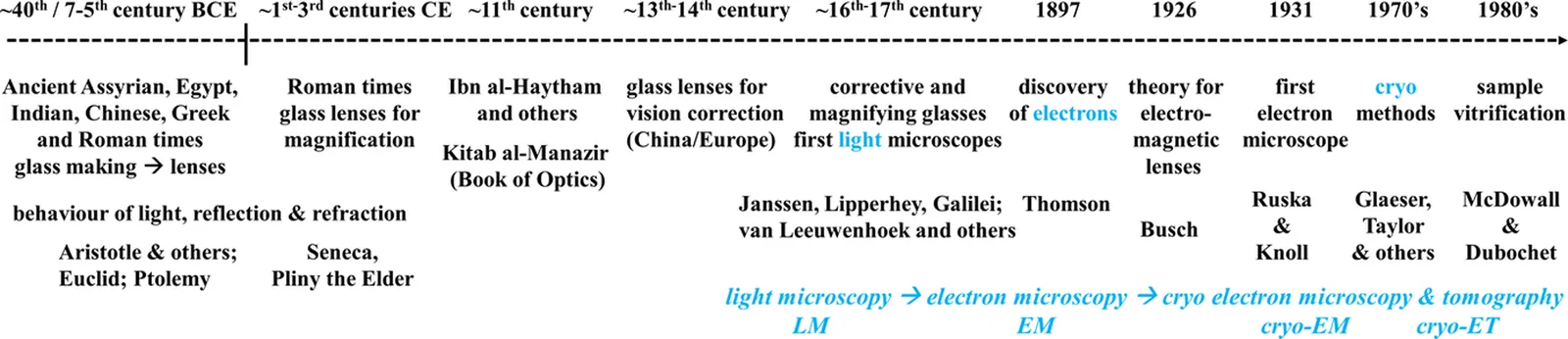
How the term ‘cryo electron microscopy’ emerged over time and along with technological developments. Early descriptions of behaviour of light and early glass lenses are reported from many centuries BCE (ancient Assyrian, Egypt, Indian, Chinese, Greek and Roman times) and evolved over long periods of time. Glass lenses were and are used with visible light (LM), but with the discovery of electrons and the use of electromagnetic lenses it became possible to built electron microscopes, resulting in EM. Cryo developments then allowed better preservation of biological specimens, thus giving rise to cryo electron microscopy (cryo-EM) and cryo electron tomography (cryo-ET; the central keywords are highlighted in blue). The chronological order of discoveries and developments is therefore directly relevant for the terminology used in the cryo-EM and cryo-ET fields.

The abbreviation ‘cryo-EM’ stands for ‘cryo electron microscopy’ and originally evolved from the general term ‘microscopy’ [from the ancient Greek words ‘μικρ
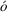
ς’ (mikrós, ‘small’) and ‘σκοπɛ
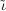
ν’ (skopeîn, ‘to look’) meaning ‘examination of small things’]. Microscopy started by using visible light [wavelength range of ∼380–750 nm; light microscopy (LM)] and glass lenses [developments in the late 16th to early 17th century attributed to Hans and Zacharias Janssen, Hans Lipperhey, Galileo Galilei, Antonie van Leeuwenhoek, and many others (corrective and magnifying glasses were also used for astronomy); for a historical overview see Fig. 2 ▸], but with the discovery of electrons (Joseph J. Thomson, 1897) the first electron microscope could be built (Ernst Ruska and Max Knoll, 1931) using electromagnetic lenses [instead of glass lenses in LM, based on work by Busch (1926 ▸)] giving rise to ‘electron microscopy’ (EM) (see https://en.wikipedia.org/wiki/Timeline_of_microscope_technology) (not ‘electronic’ microscopy as sometimes erroneously used as a result of typographical or translation errors). The wavelength of electrons depends on the acceleration voltage, *e.g.* using the de Broglie wavelength relativistic formula: λ ≃ 0.037 Å at 100 kV, 0.025 Å at 200 kV, and 0.020 Å at 300 kV; this is of the order of 50× smaller than X-rays and 250 000× smaller than visible light. Hence, EM is not wavelength-limited (diffraction-limited) for achieving Ångstrom resolution as LM is. With the development of cryo-methods to better preserve biological specimens and reduce radiation damage using frozen-hydrated specimens while working at cryo-temperatures (Robert Glaeser and Kenneth Taylor in the mid-1970s – Taylor & Glaeser, 1974 ▸; Glaeser & Taylor, 1978 ▸), and the plunge-freezing and vitrification breakthroughs (Alasdair McDowall and Jacques Dubochet, 1981–1984 – Dubochet & McDowall, 1981 ▸; Dubochet *et al.*, 1982 ▸), the word ‘cryo’ was added (Fig. 2 ▸). As ‘cryo’ is to be understood as a descriptive and specific feature of EM (as compared with the general concept of EM or LM established before), it is meant to be used as an adjective prefix, resulting in the keyword ‘cryo-EM’, which is also widely adopted by scientific journals in the structural biology field.

Fixing biological samples in vitreous ice [*i.e.* non-crystalline or amorphous ice; as some properties of liquid water are maintained, such as molecular diffusion, one could also argue for the term ‘cryo-cooled water’ as sometimes used in X-ray crystallography (Garman, 1999 ▸; Klaholz, 1999 ▸; Juers & Matthews, 2004 ▸)] has three advantages: (1) it preserves the specimen and reduces radiation damage; (2) it fixes the particles and eliminates molecular movements, thereby allowing the orientation of individual particles to be frozen; and (3) it allows the specimen to be preserved within the electron microscope, which operates at high vacuum to avoid absorption of electrons in air and reduce ice contamination. Three-dimensional reconstruction from 2D projections assumes (i) homogeneous composition and conformation of the complexes of interest, and (ii) even angular distribution of particle orientations (views). This is often not the case. Hence, specific methods have been developed to address sample heterogeneity by particle sorting and structure separation using 2D and 3D classification methods, as well as local/focused classification and refinements or multibody refinement (Klaholz *et al.*, 2004 ▸; White *et al.*, 2004 ▸; Simonetti *et al.*, 2008 ▸; Loerke *et al.*, 2010 ▸; Klaholz, 2015 ▸; Ilca *et al.*, 2015 ▸; Scheres, 2016 ▸; von Loeffelholz *et al.*, 2017 ▸; Nakane *et al.*, 2018 ▸; Zhang *et al.*, 2019 ▸; Barchet *et al.*, 2023 ▸; Vuillemot *et al.*, 2023 ▸; von Loeffelholz *et al.*, 2026 ▸); such approaches can help to separate distinct structural and functional states from heterogeneous samples [Fig. 1 ▸(*c*)], which would otherwise not be easily amenable through individual biochemical isolation. The second issue regarding preferred particle orientations can be analysed or plotted in various software [*e.g.Relion* (Scheres, 2012 ▸), *cryoSPARC* (Punjani *et al.*, 2017 ▸), *VUE* (Urzhumtseva *et al.*, 2024 ▸; Urzhumtsev, 2026 ▸), *BKPR*/*Eulerplot* (Orlov & Klaholz, 2026 ▸)] and at least in part corrected for *a posteriori* in different manners [Sorzano *et al.*, 2021 ▸; *IMAGIC* software (van Heel *et al.*, 1996 ▸), *VUE* program (Urzhumtseva *et al.*, 2024 ▸; Barchet *et al.*, 2026 ▸) or *BKPR* (Orlov & Klaholz, 2026 ▸)] through down-weighting (not ‘rebalancing’) preferential orientations or *a priori* by collecting tilted data to create a more uniform (balanced) distribution of particle orientations.

The term ‘cryogenic’ by itself implies ‘creating cold’ (Greek: ‘κρ
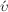
ος’ (kryos) ‘cold’ and ‘γɛν
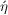
ς’ (genēs) ‘generate’), but at best this is true only for the liquid nitro­gen and ethane used to cryo-cool the samples; to avoid devitrification, the low temperature has to be maintained during sample transfer and data acquisition, but the microscope does not generate cold. Hence, cryo-EM uses but does not create cold so the term ‘cryogenic EM’ that is sometimes used is not ideal. ‘Cryo’ alone explains it well, indicating that EM working at low sample temperatures is meant.

Taken together, for reasons of historical developments and concepts and the chronological order of word evolution (Fig. 2 ▸), the suggested wording to be used is ‘cryo electron microscopy’ or ‘cryo-EM’ (not ‘electron cryo microscopy’, which would reverse the chronological order of technological developments). ‘Cryo-EM’ contains a hyphen in the abbreviation to facilitate readability, but no hyphen when written out ‘cryo electron microscopy’. If it was written as ‘cryo-electron microscopy’ it could give a false impression because the electrons used for imaging are not ‘cold’ [I have alluded to that in courses and lectures, which this paper is based on, and independently there was also a paper on this topic where a referee apparently made a similar comment (Henderson & Hasnain, 2023 ▸)].

## 3. 3D reconstruction versus ‘model’

**Figure 3:**
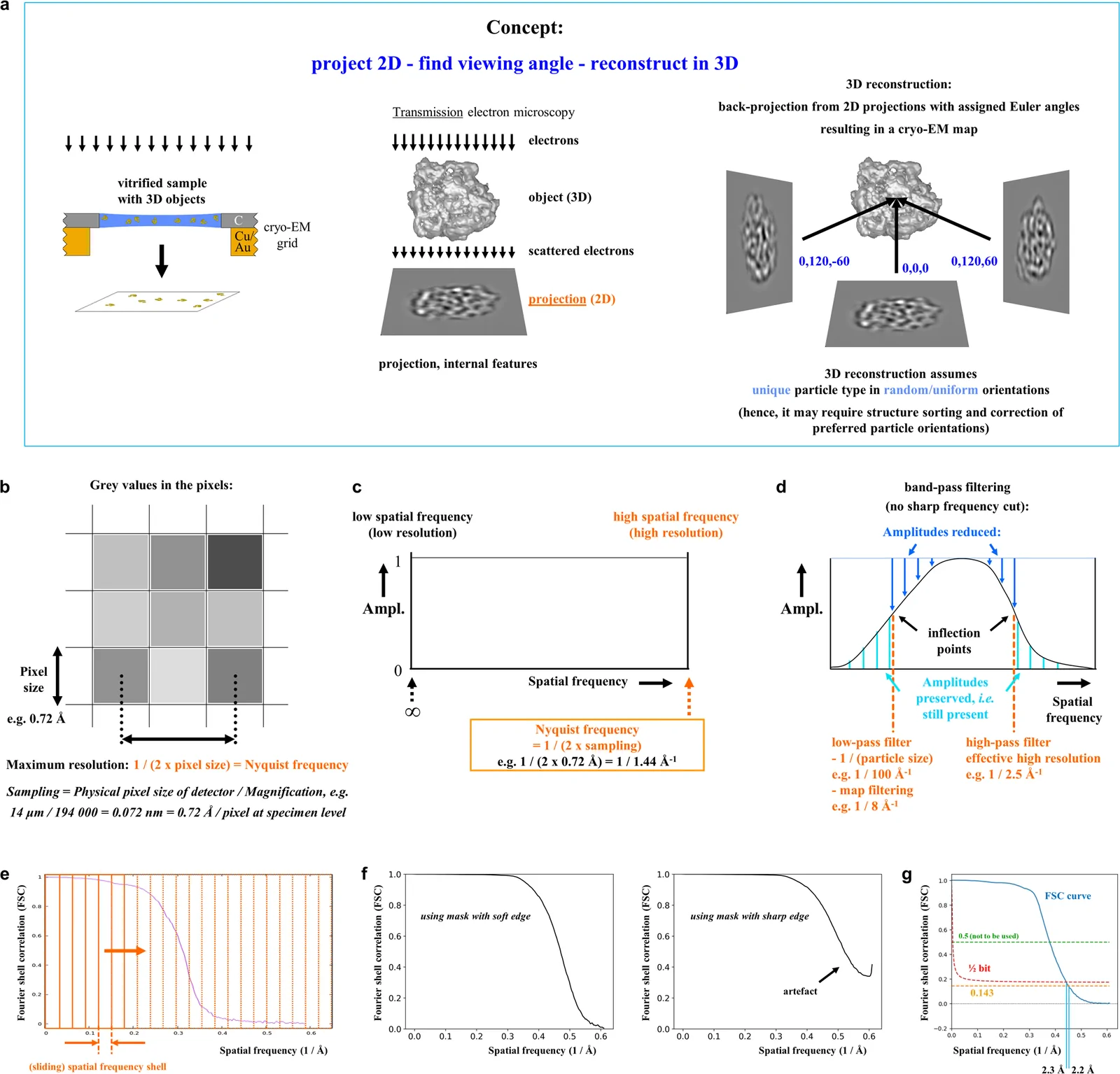
Concepts of 3D reconstruction, spatial frequency and resolution estimation in cryo-EM. (*a*) Left and middle – imaging a vitrified sample on a cryo-EM grid using electrons gives a 2D projection of the 3D object; because transmission EM is used, all internal features of the object are present in the corresponding 2D projection. Right – concept of 3D reconstruction from 2D projections using back-projection; this is the reverse process as compared with the data acquisition in the left panel. Three-dimensional reconstruction requires one to have viewing angles assigned (*i.e.* orientations of each particle view), *e.g.* as Euler angles. Three-dimensional reconstruction assumes unique particles (homogenous in terms of composition and conformation) and an equal distribution of particle orientations to avoid map distortions, otherwise appropriate measures need to be taken during image processing (*e.g.* structure sorting, focused refinements or correction of preferred particle orientations). Three-dimensional reconstruction results in a cryo-EM map that can be interpreted by building and refining an atomic model. (*b*) Relationship between the pixel size of an image and the highest spatial frequency (Nyquist frequency), which is 1/(2 times the pixel size). The sampling corresponds to the physical pixel size of the detector divided by the magnification. Resampling (or sub-sampling) reduces the number of pixels (and hence increases the pixel size) and corresponds to ‘binning’ (on a detector) and ‘coarsening’ during image processing. This is not the same as ‘rescaling’, which changes the magnification. (*c*) Reference coordinate system for a spatial frequency distribution with typical annotations; low spatial frequencies correspond to low resolution (infinitely low at the origin on the left), and high spatial frequencies correspond to high resolution (limited by the pixel size; amplitude is abbreviated as Ampl.). The unit of spatial frequency is 1/Å; hence, to calculate the resolution one needs to take the inverse value. (*d*) Example of the effect of band-pass filtering drawn into the spectrum of spatial frequencies as in (*c*) with annotation of low-pass and high-pass filter values at the corresponding inflection points. Usage of band-pass filters avoids sharp frequency cuts, which lead to artefacts in Fourier space (Fourier ripples *etc*.) during image processing. While amplitudes are reduced within the pass (boundaries within the region defined by the two inflection points), they are in part still present outside that region. (*e*) Concept of FSC calculations using thin sliding windows of spatial frequency shells within which the correlation of amplitudes is calculated between two half maps (for this the obtained image data have been split into two halves and the two corresponding 3D reconstructions were refined independently), resulting in an FSC curve [units are those defined in panel (*c*)]. (*f*) Left – FSC curve calculated using a soft-edge mask (*e.g.* 7 or 9 pixels) as is normally done. Right – FSC curve using a sharp-edge mask, which leads to correlation artefacts at high spatial frequencies and thereby shifts the FSC curve to higher values, giving the false impression of a higher resolution estimation. Using a mask *per se* is necessary to remove noise outside the core 3D reconstruction and properly evaluate the resolution of the cryo-EM map. (*g*) A typical FSC curve as can be found in the PDB validation report associated to a given EMDB cryo-EM map deposition file. The resolution estimation indicated here uses both the 0.143 threshold (Rosenthal & Henderson, 2003 ▸) and the 1/2 bit criterion (van Heel & Schatz, 2005 ▸; its numerical value asymptotically approaches 0.1716; the collected information is measured in bits per voxel in Fourier space), these often give overall comparable resolution estimation values as also visible in this example. To obtain the estimated resolution, one has to take the inverse value of the spatial frequency [as in panel (*c*)] at the intersection point. The 0.5 criterion shown in the PDB validation report here is normally used only for map–model FSC calculations and evaluation of the quality of the derived atomic model.

During cryo-EM image processing, an essential step is the 3D reconstruction that is obtained by back-projection of the input 2D projections of the particles (Harauz & van Heel, 1986 ▸), *i.e.* the reverse process of what is happening during data acquisition on a transmission electron microscope (TEM), which generates 2D projections of the 3D object embedded in the vitreous ice [Fig. 3 ▸(*a*)]. Because the sample is frozen-hydrated, all internal features of the object (*e.g.* the secondary structure of proteins) are collected in the 2D projection, as opposed to negative staining EM (note the wording, not just ‘negative stain’ or ‘neg stain’) where an envelope would be obtained from the solvent-excluded negative shape of the object. When Euler angles are assigned to the different particle orientations, a first 3D reconstruction can be made. This should be called ‘initial 3D reconstruction’ rather than the confusing term ‘initial model’ (sometimes also used in image-processing software), which in fact does not mean, as one could think, atomic models used to start interpreting maps or an initial model used for molecular replacement (in crystallography) or a ‘model’ structure to pre-align the particle images. Even when using random Euler angles or references from known structures to assign initial angles, the first low-resolution maps are initial 3D reconstructions of the object, not (initial or refined) ‘models’; this is different in the terminology of artificial intelligence, where models are indeed used: an AI model is a set of algorithms, parameters and data structures built using machine learning or deep learning techniques and trained, for example, to recognize particle images in a micrograph. With the advent of high-resolution cryo-EM (termed the ‘resolution revolution’; Kühlbrandt, 2014 ▸), map interpretation and atomic model building and refinement has become a standard approach and should not be confused with the 3D reconstruction process during image processing. Three-dimensional reconstructions are not envelopes, ‘surfaces’ or ‘volumes’, which rigorously spoken are properties measured in Å^2^ and Å^3^, respectively (yet, the term ‘volume’ is much used in cryo-ET and sometimes also in cryo-EM). The refined 3D reconstructions are called cryo-EM maps (technically, and in terms of physics, these are Coulomb potential maps or electrostatic potential maps). This is because of the nature of interactions between the electrons or photons and the atoms of the biological sample: electrons carry a negative charge and interact mostly with the nucleus of the atoms, while for X-rays the electromagnetic wave is scattered by the electron shells of the atoms (hence, maps are called ‘electron density’ maps in X-ray crystallography). The charged nature of the electrons used in cryo-EM imaging therefore also allows for gaining information on the charge distribution of residues in the biological sample, for example to localize positively charged Mg^2+^ ions (Hryc *et al.*, 2017 ▸; Wang *et al.*, 2021 ▸; Bick *et al.*, 2024 ▸).

## 4. ‘Coarsening’ versus ‘binning’, as opposed to ‘rescaling’

Another example is the word ‘binning’, often synonymously used in data processing (and software) to mean coarsening, *i.e.* sub-sampling, during image processing. Binning is what is usually done on the detector [CMOS (complementary metal oxide semiconductor) or other], which when applied on-sensor can improve the signal to noise by reducing read noise per resulting pixel, this *e.g.* helps to increase the modulation transfer function of the camera; hence, it is a data pre-processing technique that helps reduce the effects of minor observation errors. Coarsening and binning both do change the pixel size by summing values of neighbouring pixels [Fig. 3 ▸(*b*)], but they are either used during data acquisition on the camera or used later during image processing (coarsening, which is sub-sampling, can be done with non-integer values but then requires interpolation). For example, coarsening image data by 2 or 3 implies averaging 2 × 2 = 4 pixels or 3 × 3 = 9 pixels, respectively, which also changes the corresponding pixel size by 2 or 3, respectively, and reduces the number of pixels by fourfold or ninefold, respectively. With a physical pixel size of 14 µm on the direct electron detector camera, a dataset collected on a TEM instrument operating at a nominal magnification of 165 000× would result in a pixel size of 0.72 Å at the specimen level, leading to a spatial frequency of 1/1.44 Å^−1^ [according to the Nyquist–Shannon theorem, the Nyquist frequency is defined as twice the pixel size; Figs. 3 ▸(*b*) and 3 ▸(*c*)]. In this example, coarsening 2× or 3× would lead to a pixel size of 1.44 Å or 2.16 Å, respectively. Coarsening is often used in the initial steps of image processing to speed up calculations (by an approximate factor of 4× and 9×, respectively, for the example above) and is also helpful to increase image contrast, which facilitates (or even enables) particle selection, particle centring and alignment, classifications, Euler angle assignment (all of these rely mostly on low resolution/low spatial frequencies), and overall 3D reconstruction, before proceeding to structure refinement with full sampling (*i.e.* here with the original pixel of 0.72 Å). Filtering can also be useful for these cases, but works differently [in Fourier space, see principle of bandpass filtering in Fig. 3(*d*)].

In this context, upon coarsening the pixel size changes, but the absolute scale of the specimen dimensions is preserved (it does not change). This is different when ‘rescaling’ is done, for example to calibrate the magnification of the microscope, *e.g.* to fine-tune the pixel size from a nominal value of 0.72 to 0.729; this is typically done using a crystalline calibrated material (*e.g.* catalase or gold *etc*.) or cross grating grids, or by fine-scaling towards an atomic model, *e.g.* using software such as *Chimera* or *Phenix*, option map_to_model (Afonine *et al.*, 2018 ▸; Liebschner *et al.*, 2019 ▸; Klaholz, 2019 ▸; Fréchin *et al.*, 2023 ▸; Dickerson *et al.*, 2024 ▸). This has a direct influence on the value of the highest possible Nyquist frequency and hence should be considered and corrected for before doing resolution estimation of the obtained cryo-EM maps (see above). The term ‘rescaling’ is sometimes erroneously used in software to mean coarsening of the data, but the real meaning is in fact different.

## 5. Resolution estimation using Fourier shell correlation

Towards the end of the process of image processing and structure refinement (in fact map refinement, as opposed to atomic model structure refinement), the resolution of the obtained cryo-EM map is estimated by Fourier shell correlation (FSC; van Heel *et al.*, 1982 ▸; Saxton & Baumeister, 1982 ▸). The method itself is based on correlation calculations in Fourier space done for equally spaced resolution shells comprising equidistant ranges of spatial frequencies [Fig. 3 ▸(*e*)] based on two half sets of the image data. Because masks can create correlation artefacts these should be applied after overall resolution estimation and phase randomization are applied (Chen *et al.*, 2013 ▸); masks should use soft edges and must not use sharp edges, which create artefacts at high spatial frequencies [Fig. 3 ▸(*f*)], and FSC curves should not be cut so that the full spectrum out to the Nyquist frequency can be seen [Figs. 3 ▸(*e*)–3 ▸(*g*)]. As FSC serves as an average resolution estimation of a given 3D reconstruction, the value indicated should include no more than one digit after the decimal point (*e.g.* 2.7 Å, not 2.67 Å; the precision of the resolution estimation is certainly not within 0.01 Å). While different threshold criteria have been proposed [0.143 and half-bit criterion; Rosenthal & Henderson, 2003 ▸; van Heel & Schatz, 2005 ▸; Fig. 3 ▸(*g*)], another independent resolution estimation can be done by comparison with the atomic model refined into the cryo-EM map, *e.g.* the *d*_99_ value in the *Phenix* software (Afonine *et al.*, 2018 ▸). For map–model FSC calculations, the commonly used criterion is 0.5. Local resolution estimation [*e.g.* software such as *Bsoft* (Heymann & Belnap, 2007 ▸), *ResMap* (Kucukelbir *et al.*, 2014 ▸), *LocScale* (Jakobi *et al.*, 2017 ▸), *MonoRes* (Vilas *et al.*, 2018 ▸), *Phenix* (phenix_autosharpen or phenix.map_sharpening; Afonine *et al.*, 2018 ▸)] can be very useful considering that the estimated overall resolution from FSC is an average over the entire map, but structurally less ordered regions may be less resolved (in which case it can also help to filter the map to lower resolution, *e.g.* typically ∼6 Å to see protein or RNA domains). Maps obtained from cryo-EM and cryo-ET (using sub-tomogram averaging) and deposited into the EMDB (https://www.ebi.ac.uk/emdb) are required to include the overall consensus map (before classification or focused refinement if this was applied), half maps, masks and, if present, the individually refined regions that are used to assemble composite maps; validation reports (obtained from deposition of atomic coordinates to the PDB, https://www.wwpdb.org) should be provided for reviewing purposes. Raw data can also be deposited at https://www.ebi.ac.uk/empiar.

Considering that the number of structure factors (these can be obtained by Fourier transform of the cryo-EM map) increases strongly with the spatial frequency, non-linear resolution bins could also be considered (Urzhumtsev, 2025 ▸), for example using cubic scaling as is commonly done in X-ray crystallography (Fokine & Urzhumtsev, 2002 ▸; Liebschner *et al.*, 2019 ▸).

In this context, a confusing expression often heard is ‘lower’ resolution, implying a lower resolution value, but the resolution is actually better in this case. Lower resolution means worse resolution because resolution defines what can be actually resolved. At lower resolution, protein domains can be visualized, while at medium resolution, secondary structure elements can be identified (α-helices at 6–10 Å resolution, β-sheets at 4–6 Å resolution *etc*.), and at high resolution, protein side-chains and nucleic acid bases can be distinguished (*e.g.* pyrimidines versus purine); the latter typically involves a resolution range because some large residues can be seen in medium-resolution maps (*e.g.* Trp, Phe, purines *etc*.) while distinguishing fine features such as orientations of methyl and ethyl moieties of isoleucine residues needs a resolution of ∼2 Å or better [Fig. 1(*a*) ▸]. This is also the resolution range where water molecules (not just ‘waters’), ions, ion-associated water molecules [*e.g.* octahedral Mg^2+^(H_2_O)_6_ clusters and other coordinations] or chemical modification in protein post-translational modifications (*e.g.* Holvec *et al.*, 2024 ▸), DNA modifications or RNA post-transcriptional modifications can be seen (yet, independent experimental validation may be required as well).

## 6. Map interpretation: ‘contour level’ versus ‘threshold’

When cryo-EM maps are displayed in software such as *Coot* (Emsley *et al.*, 2010 ▸) or *Chimera*/*ChimeraX* (Pettersen *et al.*, 2004 ▸, 2021 ▸), it can be helpful to adjust the contour level to facilitate map interpretation and atomic model building. This implies that the map is being displayed at different sigma levels, but the map is not modified for that as opposed to ‘thresholding’ where the higher intensities are removed (this would in fact cut the map at a certain sigma level, thereby removing data information; for example, a map can be thresholded and binarized to create a mask that describes the contours of the object and helps map refinement, but this is different from the contour level concept). From experience when discussing with students, ‘lower’ and ‘higher’ contour levels are often confused expressions, but as a reminder, contouring a map at a lower contour level means using smaller sigma values and leads to seeing also the weaker intensities present (for example, to visualize a structurally less ordered region), while a higher contour level for example allows checking for the highest signal present (*e.g.* to identify a K^+^ ion or other metal ions, or checking the backbone position of a peptide or nucleic acid chain).

**Figure 4:**
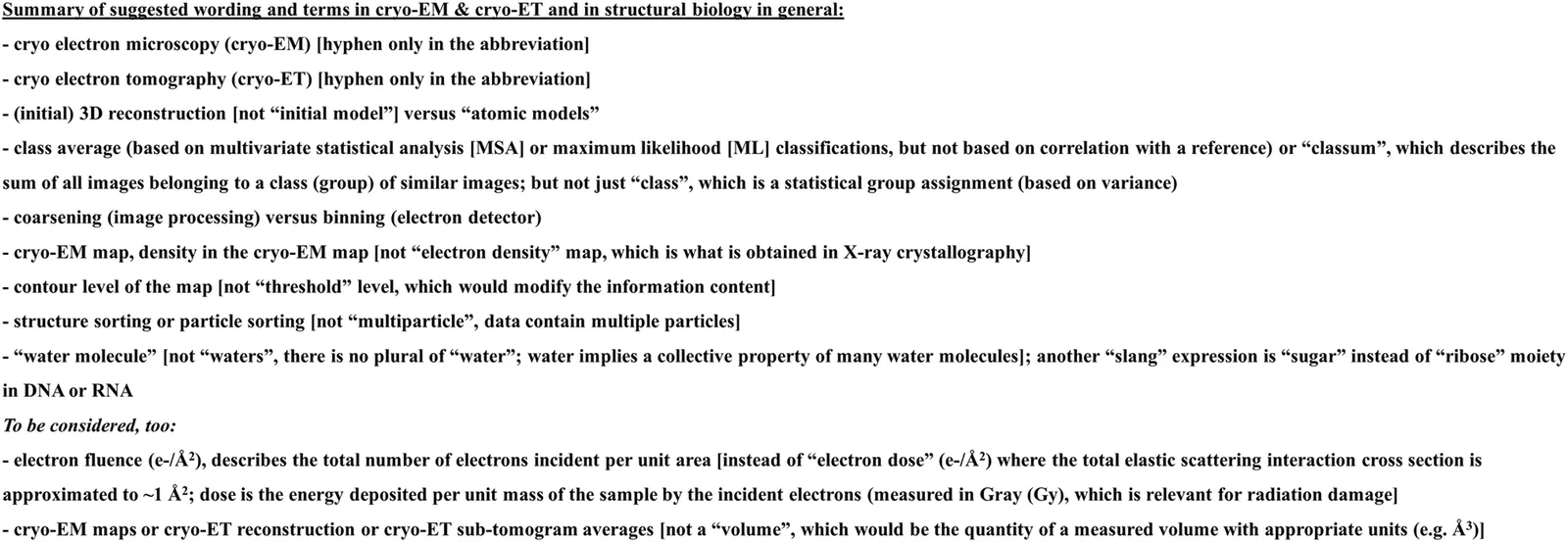
Summary of suggested terminology in cryo-EM and cryo-ET and in structural biology in general. The suggested terminology considers the consistency with the chronology of discoveries and technological developments, the underlying physical concepts, and precision of wordings to avoid confusing or misleading expressions. It is suggested to consider them in publications, within software and informatics tools therein, during courses, lectures and teaching *etc*., or in student reports and PhD theses and other manuscripts in the scientific literature.

In conclusion, this paper should be helpful for the growing community as well as for the future prospects and outreach of cryo-EM. Defining correct terms and expressions is part of the rigorous approach in science and can help everyone to use appropriate wording that conveys the same meaning (Fig. 4 ▸). There is no doubt that cryo-EM will continue to play a key role in structural biology (Fig. 1 ▸), both for high-resolution single-particle analysis (including for structure-based drug design) and for integration with multi-scale multi-resolution analysis using cellular samples [lamellae prepared using focused ion beam (FIB) devices, cryo-ET (note the consistent usage of the hyphen like in ‘cryo-EM’, also for ‘cryo-FIB’)], correlative imaging using confocal fluorescence microscopy and super-resolution imaging such as single-molecule localization microscopy.
